# Relationship between Burnout and Body Mass Index in Senior and Middle Managers from the Mexican Manufacturing Industry

**DOI:** 10.3390/ijerph15030541

**Published:** 2018-03-17

**Authors:** Oziely Daniela Armenta-Hernández, Aidé Maldonado-Macías, Jorge García-Alcaraz, Liliana Avelar-Sosa, Arturo Realyvasquez-Vargas, Miguel Angel Serrano-Rosa

**Affiliations:** 1 Department of Electric and Computational Sciences, Universidad Autónoma de Ciudad Juárez, Del Charro Ave., 450 N., Ciudad Juárez, Chihuahua 32310, México; 2 Department of Industrial and Manufacturing Engineering, Universidad Autónoma de Ciudad Juárez, Del Charro Ave., 450 N., Ciudad Juárez, Chihuahua 32310, México; amaldona@uacj.mx (A.M.-M.); jorge.garcia@uacj.mx (J.G.-A.); liliana.avelar@uacj.mx (L.A.-S.); 3 Departament of Industrial Engineering, Instituto Tecnológico de Tijuana, Calzada del Tecnológico S/N, Tijuana Baja California 22424, México; arturo.realyvazquez@tectijuana.edu.mx; 4 Departament of Psichology, Universidad de Valencia Av. de Blasco Ibáñez, 13, 46010 Valencia, España; m.angel.serrano@uv.es

**Keywords:** occupational stress, BS syndrome, normal weight, overweight, obesity

## Abstract

This research relates Burnout Syndrome (BS) with the Body Mass Index (BMI) among middle and senior managers of the Mexican manufacturing industry. Even though BS incidence is high in the Mexican industrial population, few systematic studies have explored BS and its relationship with other health problems, such as obesity. The goal of this research is to determine the relationship between BS and the BMI in employees with normal weight, overweight, and obesity. We present three structural equation models to relate BS and the BMI. The BMI ranges were determined according to the parameters (normal weight, overweight, and obesity) proposed by the World Health Organization (WHO). The sample includes 361 employees that voluntarily answered a 31-item questionnaire. We measure the levels of BS using the Maslach Burnout Inventory–General Survey (MBI-GS) and analyze anthropometric and sociodemographic data from the participants. Then, we determine the relationships between the variables through structural equation models and estimate the direct, indirect, and total effects in the three models, which show acceptable reliability. As main findings, the normal weight model has a larger explanatory power than the overweight and obesity models. The same research hypotheses were tested and the effects of BS on the BMI differ across the three models. Such results are presented by taking into account that obesity and overweight require additional factors, such as genetic factors and personal eating habits, to be better explained.

## 1. Introduction

Burnout syndrome (BS) directly affects people. It is generally defined as a feeling of deterioration, progressive exhaustion, depletion of energy, and loss of motivation. According to Freudenberger [1], BS affects overall attitudes and behaviors, which is why it is an important research topic. Most of the research conducted in Mexico explores BS and occupational stress among healthcare professionals [2,3]. Only a few studies in Mexico have been conducted to understand better BS incidence and its consequences in industrial environments. For instance, Medellín [4] explored BS in the automotive industry, whereas Aguirre, Medellín, Vázquez, Gutiérrez, and Fernández [5] studied the relationship between BS and job positions in the assembly industry. Finally, Aranda and Ibarra [6] focused on the electronics industry. 

According to Llaneza [7], the incidence of work stress and multi-causal diseases developed in the workplace is higher in developed countries. In their work, Serrano, Moya, and Salvador [8] state that occupational stress is a person’s response in the adaptive process. When this response is negative, it becomes chronic stress and eventually BS. In Mexico, BS is commonly studied among individuals whose job is to take care of others (e.g., doctors, nurses, teachers) [2], whereas the industrial population is less frequently studied. However, the degree of occupational stress suffered by industrial employees, especially middle and senior managers, presents important research opportunities. 

Studies have reported that BS is a long lasting disease that can be associated with other health problems in the industrial population [9,10,11]. Until now, the relationship between BS and other health problems is not clear, since most studies focus only on one of the three BS dimensions: professional efficacy, cynicism, or emotional exhaustion. As an example, Ahola et al. [12] found that professional efficacy directly reduces overweight, but the authors did not find any relationship between overweight and the two other dimensions of BS. As regards the manufacturing industry, few systematic studies address the relationship between BS and overweight and obesity. For instance, we only found one structural equation model that relates BS and obesity in middle and senior managers from the manufacturing industry of Ciudad Juárez [13]. Therefore, to address this limitation, this research seeks to determine the relationship between BS and the body mass index (BMI) in senior and middle managers of manufacturing industries.

## 2. Literature Review

### 2.1. Burnout Syndrome and Stress

Occupational stress refers to the physiological and psychological effects of work stressors. In 2000, BS was declared an occupational risk factor, since it affects the mental health and quality of life of workers [14]. In other words, occupational stress is the consequence of negative work conditions (including psychosocial and ergonomic conditions) that affect the health of employees. Moreover, BS is considered as the final stage of chronic occupational stress. It is a psychosocial disease related to physical complaints and diseases [15].

According to Freudenberger, BS is a state of fatigue, frustration, and loss of motivation experienced by employees whose work is to take care of others. BS results from professional activities that do not produce the expected rewards [16]. For Castillo [17] and Maslach [18], this syndrome develops through the following three dimensions: emotional exhaustion (Emo_exha), depersonalization or cynicism (CYN), and professional efficacy (Prof_Eff). 

Emotional exhaustion is a state of excessive tiredness and depletion of emotional resources. People feel emotionally exhausted since their jobs involve taking care of others constantly. Consequently, workers experience weariness, fatigue, and a series of physical and mental manifestations of emotional resource depletion [19]. Similarly, emotionally exhausted people tend to feel they have nothing left to offer. On the other hand, depersonalization is the development of cynical attitudes and negative feelings toward others. Depersonalized people experience high irritability, loss of motivation, hostility, and detachment from work. Finally, regarding professional efficacy, burned-out people feel professionally incompetent and have negative perceptions about their professional performance and the people with whom they work. Additionally, low professional efficacy affects a person’s self-esteem, productivity, and ability to tolerate pressure and maintain professional relationships. In other words, there is a discrepancy between professional expectations and reality.

Stress has traditionally been defined as a stimulus or as a physiological response that has negative effects, such as anxiety [20]. Sinha claims that stress is a process of adaptation. In turn, adaptation is the ability of people to achieve physiological stability during changes in the internal environment and maintain apparent stability at a new physiological set point [21]. Another definition of work stress refers to it as a set of emotional, cognitive, physiological, and behavioral reactions that occur in situations of excitement. Burnout is only one of the ways in which work stress can progress. 

Burnout is an occupational disease characterized by chronic mental and physical fatigue produced by a prolonged exposure to a stressful situation [22]. The difference between burnout and stress relies on the reaction mode. Stress as a positive natural response drives us to progress; however, when stress becomes a negative state, it is called burnout. Unlike stress, burnout makes us give up [11]. Stress is one of the factors that affect obesity, and stress-induced eating has received considerable attention. Nevertheless, the relationship between stress and obesity has not been fully defined, especially because it is difficult to manage both conditions [23].

### 2.2. Body Mass Index

Obesity and overweight are public health concerns that affect developed and developing countries alike. The World Health Organization (WHO) [18] considers obesity as an epidemic of a chronic, multi-causal, and non-communicative disease that usually starts at an early age. Nowadays, more than 2.3 billion people are overweight and 700 million are obese. The WHO declares overweight when a person’s weight is 10% to 20% greater than their height in centimeters. Namely, a person is overweight when their BMI ranges from 25 kg/m^2^ to 29.9 kg/m^2^; on the other hand, people are obese if their BMI is greater than 30.0 kg/m^2^. Accordingly, normal weight is a person’s weight lower than 10% in men and 15% in women, with respect to their height [19].

The main cause of obesity and overweight is an imbalance between calories consumed and calories expended. The WHO points out that some of the most important obesity- and overweight-related conditions are cardiovascular diseases, musculoskeletal disorders, and diseases of the locomotion system. There are many techniques to prevent and tackle obesity and overweight. For instance, Klein [20] proposes a change in our lifestyle, increased physical activity, and healthy eating habits.

Mexico and the United States hold the first places worldwide in adult obesity (30%), which is ten times higher than Japan and Korea [24]. Likewise, obesity is one of the leading causes of death across the globe. Almost 3.4 million adult people die from obesity and obesity-related conditions. At this rate, according to the Organization for Economic Co-operation and Development (OECD), by 2020 two out of three people will be overweight or obese. Such numbers present a threat to life expectancy worldwide [25].

### 2.3. Relationship between Work Stress, BS and BMI

The relationship between BS and chronic diseases, particularly obesity and overweight, is not sufficiently studied. Similarly, the impact of BS on a person’s BMI has not been clearly defined in the literature. To address this gap, our research seeks to comprehensively determine the relationship between BS and the BMI. 

Researchers point out that what work stress and obesity have in common is that they are physiological and behavioral responses [23]. Likewise, it has been found that occupational stress can be associated with cardiovascular diseases, diabetes, and obesity, which in turn are leading causes of death [24]. In addition, Nevanperä assures that occupational stress leads to unhealthy eating, which consequently encourages overweight and obesity. Similarly, other researchers point out that while some people might eat a lot or unhealthily as a result of stress, others on the contrary, might lose their appetite and can lose weight [26,27,28]. 

The study presented by Foss and Dyrstad indicates that occupational stress is not the only cause of obesity. Other factors, such as age, gender, and individual coping mechanisms, have a strong influence on a person’s weight [29]. Similarly, some other studies have found associations between different work stress related measures, such as cortisol in hair, with the BMI [30,31,32]. Additionally, a study using longitudinal data found that occupational stress induces weight loss in lean individuals and weight gain in overweight individuals [33]. However, the authors of that study also recognize that inconsistent results were found in previous studies [34,35,36,37].

**Figure 1:**
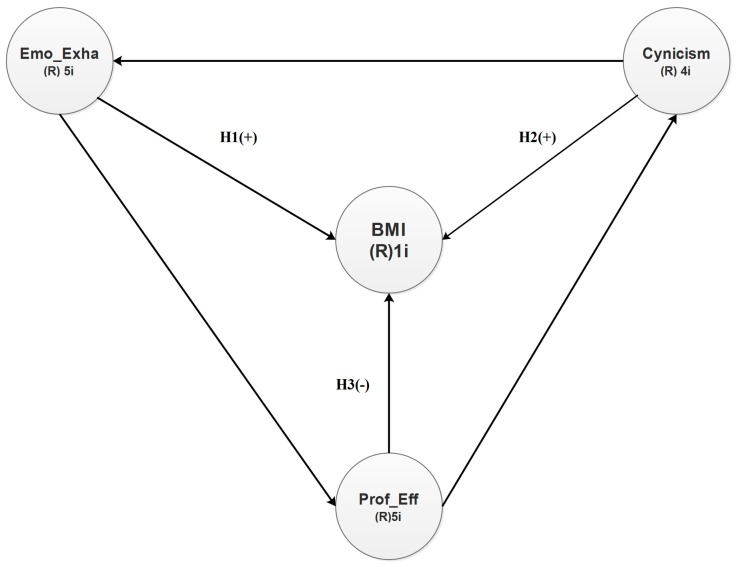
Burnout-BMI hypothetical model. H1: The emotional exhaustion (Emo_Exha) affects directly and positively the Body Mass Index (BMI); H2: The Cynicism affects directly and positively the Body Mass Index (BMI); H3: The Professional Efficacy (Prof_Eff) affects directly and negative the Body Mass Index (BMI). Emotional Exahustion (Emo_Exha) and Professional Efficacy (Prof_Eff).

The relationships between occupational stress and the BMI are far to be clear. Moreover, few studies directly analyze the relationships between the three BS dimensions and the BMI. In this sense, our hypothetical model introduced in Figure 1 establishes the relationships between the three BS dimensions and the BMI. These hypotheses can expressed as follows:

**Hypothesis 1** (H1)**.** In senior and middle managers of the manufacturing industry of Ciudad Juárez, Mexico, there is a positive relationship between cynicism and the BMI.

**Hypothesis 2** (H2)**.** There is a positive relationship between emotional exhaustion and the BMI.

**Hypothesis 3** (H3)**.** There is a negative relationship between professional efficacy and the BMI.

Notice that, in following sections, these hypotheses will be discussed and tested for each one of the three models: the normal weight model, the overweight model, and the obesity model.

## 3. Materials and Methods

### 3.1. Materials

We employed the Maslach Burnout Inventory–General Survey (MBI-GS) and a sociodemographic questionnaire to gather data and thus validate the hypotheses depicted in Figure 1.

#### 3.1.1. Maslach Burnout Inventory-General Survey

We employed the Spanish version of the MBI-GS—translated by Moreno et al. [38]—to measure the three BS dimensions. The MBI-GS is a generic version of the Maslach Burnout Inventory (MBI), since it is applicable to occupational groups other than human services providers. The 16-item MBI-GS has three subscales that parallel those of the MBI: emotional exhaustion (five items), cynicism, (five items), and professional efficacy (six items). The respondents use a seven-point Likert scale (0 = never, 1 = rarely throughout the year, 2 = occasionally throughout the year, 3 = several times throughout the year, 4 = frequently throughout the year, 5 = almost every day, 6 = every day) to answer each survey item. Two examples of the MBI-GS items are “I can efficiently solve problems at work” (item 5) and “I feel fulfilled when I work” (item 11). In this sense, low professional efficacy scores and high emotional exhaustion and cynicism scores imply the existence of BS. Various studies corroborate the structure of the three subscales [39]. 

#### 3.1.2. Sociodemographic Questionnaire

We designed a sociodemographic questionnaire to gather the necessary information to characterize the sample (e.g., gender, marital status, number of children, current job position, and seniority). Also, the respondents reported their anthropometric measures (i.e., weight and height). We followed WHO’s criteria to define each participant’s BMI. 

### 3.2. Method

This section describes the methodology followed to establish the relationships between BS and the BMI. The following subsections thoroughly describe each stage of the methodology.

#### 3.2.1. Sample Selection

To study the population, we relied on a non-probabilistic sample, selected through the snowball sampling technique. We scheduled meetings with the participating companies and administered the survey to the employees. As inclusion criteria, we took into account factors reported in the literature with respect to BS and its health consequences. In addition, we took into account relevant characteristics of the research population, yet studies about manufacturing industry managers are scarce. To administer the survey, we presented the research project to the healthcare committee of the Manufacturing Companies Association (AMAC Index, by its Spanish acronym) located in Ciudad Juárez. The committee members are healthcare professionals that work in manufacturing companies. Those committee members who were interested in the research helped us reach the companies where they work.

#### 3.2.2. Fieldwork and Survey Administration

The survey had two sections: the MBI-GS and the sociodemographic questionnaire. It was administered in six manufacturing companies located in Ciudad Juárez, Mexico. Three of the surveyed companies are automotive manufacturers, two are electrical manufacturers, and one produces miscellaneous products. Depending on its availability, each company scheduled a meeting for us to administer the survey.

#### 3.2.3. Database Construction and Screening

Using software SPSS^®^ v.20 (IBM company, Chicago, IL, USA), we built a database with the data collected from the 361 participants. Then, the data were classified according to each respondent’s BMI. Next, we screened the database to detect missing values and outliers. Because the MBI-GS data were ordinal, the missing values were replaced with the median. On the other hand, because the BMI is a continuous variable, its missing values were replaced with the mean. As for the outliers, we constructed box-and-whisker plots to detect them and prevent data input errors. Outliers are usually unanswered items and occur when participants do not know how to answer a question or do not want to respond to it. We replaced missing values with the median value of the items in all the questionnaires, because they all had less than 10% of missing values. Namely, an ordinal missing value was replaced with the median value of the item, and an interval or numeric missing value was replaced by the mean [40].

#### 3.2.4. Statistical Validation of Data

Some quality and reliability coefficients were estimated to validate the reliability of the administered questionnaires. Namely, we estimated the Cronbach’s alpha and the composite reliability index.

#### 3.2.5. Descriptive Analysis of the Sample

Using software SPSS^®^ v.20, we performed a descriptive analysis of the sample. This analysis highlighted and related the sociodemographic variables (i.e., gender, academic level, marital status, type of contract, seniority, work schedule, job position, and department).

#### 3.2.6. Generation of Structural Equation Models

Structural Equation Modeling (SEM) is a statistical analysis technique that can detect all the relationships between variables and test pre-established hypotheses. The SEM-based analysis was conducted on WarpPLS (ScriptWarp Systems, Laredo, TX, USA), which uses the partial least squares (PLS) method to analyze data, create models with non-linear relationships, and generate only unidirectional relationships [41,42]. Moreover, PLS allow researchers to work with non-normal data and small samples. The goal of the PLS method is to explain the variance of the created constructs and thus minimize the error and the explained variance. Finally, the PLS method enables researchers to build predictive models when the factors are many and highly collinear [41,43,44].

At this stage, we developed and validated the three models using SEM. Similarly, we defined the model variables as follows: Emo_Exha (emotional exhaustion), CYN (cynicism), Prof_Eff (professional efficacy), and BMI (body mass index). Then, using WarpPLS v 6.0 (ScriptWarp Systems, Laredo, TX, USA), we analyzed the relationships between these latent variables with respect to the three research hypotheses. The criterion for accepting or rejecting a hypothetical relationship was the *p* value.

##### Model Fit Indices

We estimated the Cronbach’s alpha and the composite reliability index to measure the internal reliability of each latent variable, setting 0.7 as the minimum acceptable value. Additionally, seven latent variable coefficients were estimated: R-Squared (R^2^), Q-Squared (Q^2^), Adjusted R-Squared, the Composite reliability index, VIF, and AFVIF. On the other hand, we computed AVE as a measure of convergent validity, setting 0.5 as the minimum acceptable value. To measure predictive validity, we estimated the R^2^ coefficient and the Q^2^ coefficient. Experts recommend accepting R^2^ values higher than 0.02 and Q^2^ values higher than 0 and similar to their corresponding R^2^ values. Additionally, we estimated the variance inflation factors (VIF) and the average variance inflation factor (AVIF) to measure internal collinearity. In this research, only values lower than 5 were acceptable. Finally, eight model fit and quality indices proposed by Knock were estimated for every model: Average Path Coefficient (APC), Average R-Squared (ARS), Average Adjusted R-Squared (AARS), Average Block VIF (AVIF), Average Full collinearity VIF (AFVIF), and the Tenenhaus Goodness of Fit (GoF, criteria > 0.36) [30]. The Table 1 shows the criteria for the indicators.

##### Determination of Direct and Indirect Effects

All the effects between the latent variables were analyzed using WarpPLS 4.0© (ScriptWarp Systems, Laredo, TX, USA). In SEM, direct effects are usually depicted with arrows that directly connect two latent variables. The direct effects helped us validate the research hypotheses (see Section 2.3). The decomposition of direct effects was also performed at this stage with respect to the R^2^ values. The goal was to analyze the percentage of explained variance from each independent latent variable. Finally, we also calculated the sum of indirect effects. Indirect effects between two latent variables occur through paths with two or more model segments. The sum of all the indirect effects in a relationship corresponds to the total indirect effects for that relationship. Finally, we analyzed total effects. Total effects in a relationship are the sum of the direct and indirect effects.

## 4. Results

This section presents the results of the sample’s descriptive analysis and those obtained from the three models: the normal weight model, the overweight model, and the obesity model. 

### 4.1. Sample Characteristics

In total, 361 senior and middle managers from six manufacturing companies located in Ciudad Juárez, Chihuahua, Mexico, were surveyed. The surveyed job positions included chief executives, supervisors, group leaders, engineers, and administrative staff. The participants were classified in three groups according to their BMI: normal weight, overweight, and obesity. The sample included 69% men and 31% women. Table 2 shows some of the sample’s main characteristics.

### 4.2. Latent Variable Validation and Model Efficiency—Normal Weight Model

Table 3 shows the latent variable coefficients for the normal weight model. As can be observed, the Cronbach’s alpha and the composite reliability values are higher than 0.7, the threshold. Similarly, all the AVE values are above 0.5, which confirms the survey’s discriminant validity. Likewise, because all the VIF values are lower than 3.3, we conclude that there are no collinearity problems between the latent variables [31]. Finally, all the Q^2^ values seem to be higher than 0 and similar to their corresponding R^2^ values. Such results confirm that the latent variables have acceptable parametric validity.

### 4.3. Model Fit Indices—Normal Weight Model

The model fit indices presented in Table 4 demonstrate that the normal weight model is efficient, since all the *p* values are lower than 0.05. In addition, the results demonstrate that the model has good predictive power and good explanatory power, the latter according to the GoF index. In conclusion, the normal weight model is efficient.

### 4.4. Direct effects—BS and BMI for Normal Weight Model

**Figure 2:**
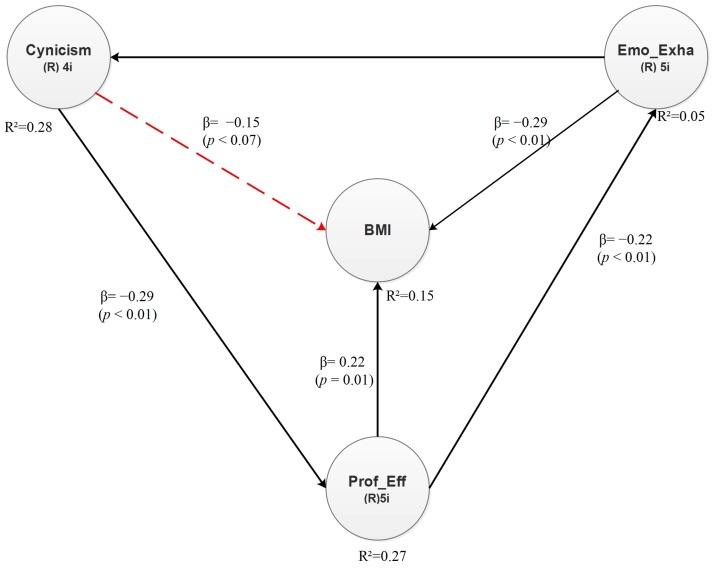
Direct effects—Normal weight model.

Figure 2 illustrates the direct effects between the latent variables. Every direct effect is associated with a beta (β) value and a *p* value. The former indicates dependency in standard deviations, whereas *p* is the significance value from the hypothesis test. Significant relationships have a *p* value lower than 0.05, and consequently, they are statistically significant at a 95% confidence level. As the model indicates, the direct relationships between Emotional Exhaustion and Normal Weight (BMI) and between Professional Efficacy and Normal Weight (BMI) are significant, whereas the direct relationship between Cynicism and Normal Weight (BMI) is not significant (see dotted arrow).

Hypotheses and structural equations for the normal weight model:

**Hypothesis 4** (H4)**.** In senior and middle managers of the manufacturing industry of Ciudad Juárez, Mexico, there is a positive relationship between cynicism and normal weight.

**Hypothesis 5** (H5)**.** There is a positive relationship between emotional exhaustion and normal weight.

**Hypothesis 6** (H6)**.** There is a negative relationship between professional efficacy and normal weight.

Considering the direct effects between the latent variables, the structural equation for dependent latent variable Normal Weight (BMI) can be proposed as follows:

Normal Weight (BMI): −0.29 × Emotional Exhaustion + 0.22 × Professional Efficacy + Error(1)

The conclusions with respect to the R^2^ values are presented in Table 5. Notice that 5% of the variance of Emotional Exhaustion is explained by Professional Efficacy, while 25.8% of the variance of Cynicism can be explained by Emotional Exhaustion. Likewise, Cynicism explains 27% of the variance of Professional Efficacy. Finally, Emotional Exhaustion and Professional Efficacy together explain 14.6% of the variance of Normal Weight (BMI).

### 4.5. Sum of Indirect Effects—Normal Weight Model

Relationships in SEM are not always direct. Table 6 presents the sum of the indirect effects for the normal weight model. Some of these effects have *p* values greater than 0.05, which implies that such indirect relationships are not statistically significant. On the other hand, one of the most notable indirect relationships involves latent variables Cynicism and Normal Weight (BMI). In this relationship, β = −0.147 implies that when the first latent variable increases by one standard deviation, the second latent variable decreases by 0.147 standard deviations. Consequently, Cynicism can explain 7% of the variability of Normal Weight (BMI). Likewise, in the relationship between Emotional Exhaustion and Professional Efficacy, β = −0.277 implies that when Emotional Exhaustion increases by one standard deviation, Professional Efficacy decreases by 0.277 standard deviations. Consequently, Emotional Exhaustion can explain 6.7% of the variability of Professional Efficacy. Finally, in the relationship between Professional Efficacy and Cynicism, if the former increases by one standard deviation, the latter decreases by 0.119 standard deviations (β = −0.119). Consequently, Professional Efficacy can explain 7% of the variability of Cynicism. The remaining significant relationships can be similarly interpreted.

### 4.6. Total Effects—Normal Weight Model

Table 7 introduces the total effects found in the normal weight model. As can be observed, some of these effects are not statistically significant at a 95% confidence level. In other words, their corresponding *p* values are higher than 0.05. On the other hand, the highest total effects occur in the relationship between Professional Efficacy and Emotional Exhaustion. When Professional Efficacy increases by one standard deviation, Emotional Exhaustion decreases by 0.224 standard deviations. Consequently, Professional Efficacy explains 9.6% of the variability of Emotional Exhaustion. Similarly, the table shows that when Professional Efficacy increases by one standard deviation, Normal Weight (BMI) increases by 0.283 standard deviations. In this sense, Professional Efficacy explains up to 9.5% of the variability of Normal Weight (BMI). The remaining relationships can be similarly interpreted.

### 4.7. Latent Variable Validation—Overweight Model

Table 8 introduces the latent variable coefficients for the overweight model. Note that the Cronbach’s alpha and the composite reliability index show values higher than 0.7 in all the latent variables. Additionally, all the values of AVE are above 0.5. This confirms the survey’s discriminant validity. In addition, VIF is lower than 3.3 in all the latent variables, which discards collinearity problems between them [31]. Finally, Q^2^ values are all higher than 0 and similar to their corresponding R^2^ values. Such results confirm that all the latent variables have acceptable values in the parametric test. This model was tested at a 90% confidence level.

### 4.8. Model Fit Indices—Overweight Model

As Table 9 indicates, all the *p* values are lower than 0.10. In addition, the results prove that the model has good predictive power and good explanatory power, the latter according to the GoF index. In conclusion, the overweight model is efficient.

### 4.9. Direct Effects—Overweight Model

The hypotheses for the overweight model can be proposed as follows:

**Hypothesis 7** (H7)**.** In senior and middle managers of the manufacturing industry of Ciudad Juárez, Mexico, there is a positive relationship between cynicism and overweight.

**Hypothesis 8** (H8)**.** There is a positive relationship between emotional exhaustion and overweight.

**Hypothesis 9** (H9)**.** There is a negative relationship between professional efficacy and overweight.

**Figure 3:**
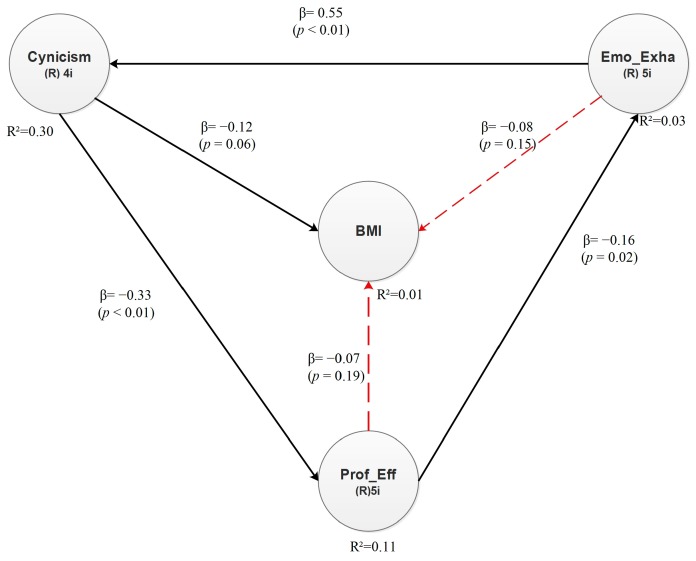
Direct effects—Overweight model.

The effects found in this model are statistically significant at a 90% confidence level. They are introduced in Figure 3. Every direct effect includes a β value and a *p* value. The former expresses dependency in standard deviations, whereas *p* is the significance value from the hypothesis test. Significant relationships have a *p* value lower than 0.10 and are statistically significant at a 90% confidence level. The only relationship that is directly significant involves latent variables Cynicism and Overweight, whereas the relationships between Emotional Exhaustion and Overweight and between Professional Efficacy and Overweight are not significant (see dotted arrows). The conclusions with respect to the R^2^ values are introduced in Table 10.

Note that Emotional Exhaustion is the only latent variable that has an impact on Cynicism. It explains 29.8% of the variability. Additionally, 11% of the variability of Professional Efficacy is explained by Cynicism. Cynicism also explains 1.4% of the variability of Overweight (BMI).

Considering the direct effects between the latent variables, the structural equation for dependent latent variable Overweight (BMI) can be proposed as follows:

Overweight (BMI) = −0.12 × Cynicism + Error(2)

### 4.10. Sum of Indirect Effects—Overweight Model

Table 11 shows the indirect effects found in the overweight model. Some of them have a *p* value higher than 0.10, which implies that such indirect relationships are not statistically significant. On the other hand, one of the most relevant indirect effects involves latent variables Cynicism and Overweight (BMI). According to our findings, when Cynicism increases by one standard deviation, Overweight (BMI) decreases by 0.065 standard deviations. Similarly, Cynicism can explain 0.7% of the variability of the BMI (Overweight).

### 4.11. Total Effects—Overweight Model

Table 12 reports the total effects found in the overweight model. Some of them are not statistically significant at a 90% confidence level, since the *p* values are higher than 0.10. However, some of the most relevant total effects occur in the relationship between Emotional Exhaustion and Cynicism. When Emotional Exhaustion increases by one standard deviation, Cynicism increases by 0.546 standard deviations. Consequently, Emotional Exhaustion explains up to 29.8% of the variability of Cynicism. Additionally, in the relationship between Cynicism and Professional Efficacy, when the former increases by one standard deviation, the latter decreases by 0.333 standard deviations. Consequently, Cynicism can explain 11.1% of the variability of Professional Efficacy.

### 4.12. Obesity Model

**Figure 4:**
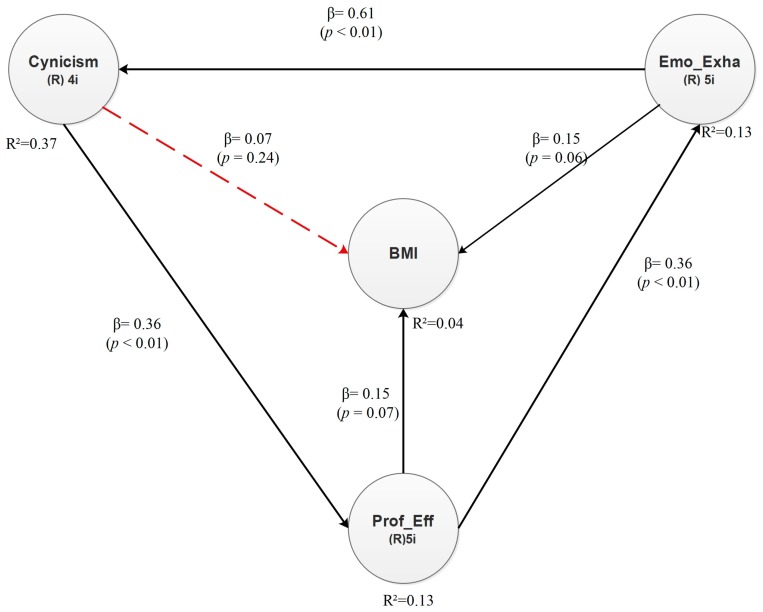
Direct effects—Obesity model.

This subsection discusses the results found in the obesity model. The effects found in this model are statistically significant at a 90% confidence level. They are depicted in Figure 4. 

### 4.13. Latent Variable Validation—Obesity Model

Table 13 shows the latent variable coefficients for the obese model. All the values of the Cronbach’s alpha and the composite reliability index are higher than 0.7, the threshold. Similarly, AVE values are all higher than 0.5, which confirms the survey’s discriminant validity. As for VIF, its value is lower than 3.3 in all the latent variables. This proves that the model is free from collinearity problems [31]. Finally, all the Q^2^ values are higher than 0 and similar to their corresponding R^2^ values. These results prove that the latent variables have acceptable values in the parametric test. 

### 4.14. Model Fit Indices

The model fit indices presented in Table 14 demonstrate that the obesity model is efficient, since all the *p* values are lower than 0.10. Likewise, the results prove that the model has good predictive power in all its parameters and good explanatory power, the latter according to the GoF index. In conclusion, the obesity model is efficient.

### 4.15. Direct Effects—Obesity Model

The direct effects found in the obesity model are depicted in Figure 4. As can be observed, all the latent variables, except Cynicism, have direct effects on the dependent latent variable.

The hypotheses for the obesity model can be proposed as follows:

**Hypothesis 10** (H10)**.** In senior and middle managers of the manufacturing industry of Ciudad Juárez, Mexico, there is a positive relationship between cynicism and obesity (BMI).

**Hypothesis 11** (H11)**.** There is a positive relationship between emotional exhaustion and obesity (BMI).

**Hypothesis 12** (H12)**.** There is a negative relationship between professional efficacy and obesity (BMI).

Every direct effect has a β value and a *p* value. The former expresses dependency in standard deviations, whereas *p* is the significance value from the hypothesis test. Significant relationships have a *p* value lower than 0.10, and therefore, they are statistically significant at a 90% confidence level. The results depicted in Figure 4 confirm that the relationships between Emotional Exhaustion and Obesity (BMI) and between Professional Efficacy and Obesity (BMI) are directly significant, whereas the relationship between Cynicism and Obesity (BMI) is not significant (see dotted arrow). As for the effect sizes reported in Table 15, we found that Professional Efficacy explains 12.9% of the variability of Emotional Exhaustion, whereas Emotional Exhaustion explains 37% of the variability of Cynicism. Notice that this is the largest direct effect for the obesity model. Finally, we found that Cynicism explains 13.2% of the variability of Professional Efficacy, while Emotional Exhaustion and Professional Efficacy respectively explain 2.2% and 2.1% of the variability of Obesity (BMI).

Considering the direct effects between the latent variables, the structural equation for the dependent variable BMI in the Obesity model can be proposed as follows:

Obesity (BMI) = 0.15 × Emotional Exhaustion + 0.15 × Professional Efficacy + Error(3)

### 4.16. Sum of Indirect Effects—Obesity Model

Table 16 shows the sum of indirect effects for the obesity model. Only those effects with a *p* value lower than 0.10 are statistically significant at a 90% confidence level. For instance, in the relationship between Professional Efficacy and Cynicism, when Professional Efficacy increases by one standard deviation, Cynicism increases by 0.219 standard deviations. Moreover, the former explains 7% of the variability of the latter.

### 4.17. Total Effects—Obesity Model

Table 17 reports the total effects estimated for the obesity model. Some of these effects are not statistically significant at a 90% confidence level, since their corresponding *p* values are higher than 0.10. On the other hand, the largest total effects (with *p* < 0.001) were found in the relationship between Emotional Exhaustion and Cynicism and imply that when Emotional Exhaustion increases by one standard deviation, Cynicism increases by 0.609 standard deviations. Consequently, Emotional Exhaustion explains up to 8.6% of the variability of Cynicism. Similarly, in the relationship between Cynicism and Professional Efficacy, when the first latent variable increases by one standard deviation, the second latent variable decreases by 0.364 standard deviations. Additionally, Cynicism explains up to 9.2% of the variability of Professional Efficacy.

## 5. Discussion

Following the literature review presented in the first section, and the results obtained after analyzing the models, it is possible to compare our findings with those reported in previous studies. According to Proper et al. [32], emotional exhaustion is associated with binge eating as a result of stress. In turn, binge eating contributes to overweight and obesity. Additionally, Montiel [45] argues that industrial workers and overall top positions tend to skip meals or eat unhealthy food, such as snacks and soft drinks, yet these foods frequently contribute to feelings of anxiety and stress. Our findings, along with those from the aforementioned researchers, help us understand better the behavior of our sample. Even though our findings differ from those reported in other studies, they help us understand the relationships between BS and overweight and obesity. 

Researchers such as Ahola [12] and Camacho et al. [9] state that professional efficacy (Prof_Eff) is directly associated with weight loss in physically active people, yet emotional exhaustion encourages obesity and overweight. The more tired people feel, the less healthy they eat. From a different perspective, we reviewed studies on BS in order to find out why burnout rarely or never explains the variability of the BMI in terms of overweight and obesity. Then, we concluded that, in such cases, aspects other than psychosocial factors could have more significant effects. For instance, a lack of physical activity, an unhealthy diet, genetic factors, and sleep disorders might have larger explanatory power than BS alone. On the other hand, in the case of people with normal weight, BS (and perhaps other psychosocial variables) can have a more prominent role.

As regards the results from the three models, we found that in the normal weight model, the relationship between emotional exhaustion and the BMI is negative; that is, the more exhausted people feel, the less healthy they eat. On the other hand, we found that professional efficacy can be positively associated with the BMI, since the more efficient workers feel, the more physically active they are. As for the overweight model, its relationships are more difficult to explain since other factors also contribute to overweight. However, the model demonstrates that cynicism and the BMI have a significant relationship. Consequently, it is fair to think that cynical people adopt risky behaviors (e.g., binge eating, meal skipping, fast food intake, disregard toward one’s heath) that compromise their health and thus their BMI.

Our study is also consistent with Luckhaupt [46], who concluded that office workers, administrative staff, architects, and engineers are more likely to suffer from overweight and obesity. On the other hand, according to Perea [47], middle managers have greater levels of perceived stress than senior managers, since they work more hours and must meet their superiors’ expectations. Finally, although our models reported acceptable quality, both BS and obesity are diseases of multifactorial origin. Foss [34] claims that stress can be a cause but also a consequence of obesity. Usually, obesity is considered as a consequence, since it influences many diseases, such as stress. However, stress as a cause refers to a person’s physiological adaptation to stressful situations.

Our findings demonstrate that BS loses explanatory power as the BMI increases. To some extent, psychosocial factors such as BS have a negative impact on a person’s health, yet other aspects, such as lifestyle preferences (eating habits, physical activity) and genetic factors, have a greater impact on the BMI when it reaches overweight and obesity levels. It can be argued that psychosocial factors and individual perceptions can rapidly trigger risk behaviors. Therefore, because people with normal weight can easily develop overweight and obesity in the future, it is important to provide enough preventive support while they have a normal weight [48,49]. Unfortunately, overweight and obese people have major obstacles to overcome. Both conditions produce side effects, and many overweight- and obesity-related illnesses are irreversible. In this sense, our findings support the generation of more efficient strategies for overweight and obesity prevention [50]. Manufacturing companies must concentrate economic, organizational, and human capital resources to prevent the incidence of psychosocial factors that compromise a healthy lifestyle.

## 6. Conclusions

The goal of this research was to find the relationships between the three BS dimensions and the BMI in a sample of individuals with normal weight, overweight, and obesity. The sample included senior and middle managers of the manufacturing industry from Ciudad Juárez, Chihuahua, Mexico. The three models here presented have their own results and conclusions. The normal weight model shows larger explanatory power than the other two models. Namely, the three independent latent variables can explain 15% of the variability of the dependent variable. On the other hand, the overweight model shows an explanatory power of 1%, whereas in the obesity model, the variability of the dependent latent variable is explained in 4%. The conclusions of the three models are presented below with respect to the research hypotheses:

H1: In senior and middle managers of the manufacturing industry of Ciudad Juárez, Mexico, there is a positive relationship between cynicism and the BMI.

Normal weight model: There is no significant relationship between cynicism and normal weight.

Overweight model: There is enough statistical evidence to demonstrate that this relationship is direct and positive, since when cynicism increases by one standard deviation, overweight increases by 0.12 standard deviations.

Obesity model: There is no significant relationship between cynicism and obesity.

H2: In senior and middle managers of the manufacturing industry of Ciudad Juárez, Mexico, there is a positive relationship between emotional exhaustion and the BMI.

Normal weight model: There is enough statistical evidence to declare that this relationship is negative, since when emotional exhaustion increases by one standard deviation, normal weight decreases by 0.29 standard deviations.

Overweight model: There is no positive or negative relationship between emotional exhaustion and overweight.

Obesity model: There is enough statistical evidence to demonstrate that this relationship is positive, since when emotional exhaustion increases by one standard deviation, obesity increases by 0.15 standard deviations.

H3: In senior and middle managers of the manufacturing industry of Ciudad Juárez, Mexico, there is a negative relationship between professional efficacy and the BMI.

Normal weight model: There is enough statistical evidence to demonstrate that this relationship is positive, since when professional efficacy increases by one standard deviation, normal weight increases by 0.22 standard deviations.

Overweight model: There is no significant relationship between professional efficacy and overweight.

Obesity model: There is enough statistical evidence to demonstrate that this relationship is negative, since when professional efficacy increases by one standard deviation, the BMI increases by 0.15 standard deviations.

The results of these hypotheses are consistent with other works. Cynicism is characterized by a lack of motivation and interest and the desire to get away from people. The normal weight model and the obesity model support Ahola’s [12] claim as regards the apparently inexistent relationship between cynicism and obesity. However, the overweight model developed in this research does provide evidence of the relationship between cynicism and the BMI. Similarly, in their study, Frances found a small relationship between cynicism and cholesterol levels in overweight individuals [51]. 

As for emotional exhaustion, which is characterized by feelings of physical and mental weariness, the normal weight model and the obesity model report a positive direct relationship between emotional exhaustion and the BMI. Additionally, previously conducted studies have reported that uncontrolled food intake can be a consequence of negative emotions [31,45]. As in Kivimäki [33], the overweight model does not indicate any relationship between emotional exhaustion and overweight; however, Dallman claims that overweight individuals can experience food intake alterations [52]. Finally, as regards professional efficacy (i.e., personal fulfillment, competence, and work satisfaction), the normal weight model and the obesity model report a negative relationship between this BS dimension and the BMI. These findings are consistent with those reported by Ahola, who concluded that being professionally effective reduces obesity [12].

As its main contributions, this research describes the relationships between the three BS dimensions and the BMI. The structural equation models here proposed illustrate the behavior of BS with respect to the BMI. As previously mentioned, the explanatory power of each model loses strength as the BMI increases, since other factors can be responsible for obesity and overweight. This research can support the idea of a gateway to future research. As a final conclusion, we can confirm that stress and obesity are interrelated; however, as mentioned by Koch, other factors must be explored to define clearer relationships [52].

### 6.1. Industrial Implications

If properly disseminated, our findings can encourage manufacturing companies to address the problems of obesity and overweight from a more comprehensive approach, thereby taking into account the seriousness of occupational stress and its side effects. Accordingly, government, occupational, and public health institutions will be able to gather valuable information on occupational health problems among Mexican manufacturing industries. Similarly, our findings are expected to encourage companies to propose strategies for human and organizational development in order to prevent and reduce work stress, overweight, and obesity.

### 6.2. Future Research

The results of this research pave the way for new causal research on overweight and obesity. As future work, we will seek to integrate other factors into the models, including physical activity and eating habits. The goal of refining the models would be to provide a better and sounder understanding of the variance of the BMI.

## Figures and Tables

**Table 1:** Model fit index criteria.

Indicators	Measure	Criteria
*Average Path Coefficient (APC)	Relationship significance	*p* < 0.05 or *p* < 0.10
*Average R-Squared (ARS)	Predictive validity	*p* < 0.05 or *p* < 0.10
*Average Adjusted R-Squared (AARS)	Predictive validity	*p* < 0.05 or *p* < 0.10
Average block (AVIF)	Collinearity	<5
Average full collinearity VIF (AFVIF)	Collinearity	<5
Tenenhaus (GoF)	Data fit	>0.36

* presents the values of acceptance and what each of the indicators measures.

**Table 2:** Characteristics of the Sample.

Characteristics	Normal Weight	Overweight	Obesity
Gender (M = Male, F = Female)	M = 61.1% F = 38.9%	M = 72.9% F = 27.1%	M = 69.8% F = 30.2%
Marital status	49.5% single	57.1% married	65.6% married
Academic level	61.1% bachelor’s degree	65.9% bachelor’s degree	56.3% bachelor’s degree
Weight (Kilograms, kg)	Average = 67.2 kg Minimum = 45 kg Maximum =82 kg	Average = 80.54 kg Minimum = 56 kg Maximum = 100 kg	Average = 95.23 kg Minimum = 71 kg Maximum = 115 kg
Height (Meters, m)	Average = 1.70 m Minimum = 1.50 m Maximum = 1.86 m	Average = 1.71 m Minimum = 1.49 m Maximum = 1.90 m	Average = 1.70 m Minimum = 1.46 m Maximum = 1.90 m
Age (Years)	Average = 32.7 Minimum = 19 Maximum = 57	Average = 37.65 Minimum = 18 Maximum = 60	Average = 40.27 Minimum = 24 Maximum = 60
Total sample	95	170	96

**Table 3:** Latent variable validation—Normal weight model.

Indices	Cynicism (CYN)	Emotional Exhaustion (Emo_Exha)	Professional Efficacy (Prof_Eff)	Normal Weight (BMI)
R-Squared	0.284	0.05	0.27	0.146
Adj. R-Squared	0.276	0.04	0.262	0.127
Composite Reliability	0.939	0.941	0.919	1
Cronbach’s Alpha	0.913	0.922	0.893	1
Average Variance Extracted	0.794	0.762	0.654	1
Full Collinearity VIFs	1.657	1.342	1.344	1.09
Q-Squared	0.288	0.056	0.27	0.153

**Table 4:** Model fit indices—Normal weight model.

Model Fit Index	Value	Decision Criteria
Average Path Coefficient (APC)	0.358	*p* < 0.001
Average R-Squared (ARS)	0.188	*p* = 0.014
Average Adjusted R-Squared (AARS)	0.176	*p* = 0.019
Average Block VIF (AVIF)	1.009	Acceptable if ≤ 5, ideally ≤ 3.3
Average Full collinearity VIF (AFVIF)	1.358	Acceptable if ≤ 5, ideally ≤ 3.3
Tenenhaus GoF (GoF)	0.388	Small ≥ 0.1, medium ≥ 0.25, large ≥ 0.36

**Table 5:** Effects size—Normal weight sample.

TO	FROM		
	Cynicism (CYN)	Emotional Exhaustion (Emo_Exha)	Professional Efficacy (Prof_Eff)
Cynicism (CYN)		0.258	
Emotional Exhaustion (Emo_Exha)			0.05
Professional Efficacy (Prof_Eff)	0.27		
Normal Weight (BMI)		0.093	0.053

**Table 6:** Sum of indirect effects—Normal weight model.

TO	FROM		
	Cynicism (CYN)	Emotional Exhaustion (Emo_Exha)	Professional Efficacy (Prof_Eff)
Cynicism (CYN)			β = −0.119 *p* = 0.046 ES = 0.07
Emotional Exhaustion (Emo_Exha)	β = −0.116 *p* = 0.05 ES = 0.070		
Professional Efficacy (Prof_Eff)		β = −0.277 *p* ˂ 0.001 ES = 0.067	
Normal Weight (BMI)	β = −0.147 *p* = 0.019 ES = 0.07	β = −0.06 *p* = 0.153 ES = 0.058	β = 0.066 *p* = 0.178 ES = 0.071

**Table 7:** Total effects—Normal weight model.

TO	FROM		
	Cynicism (CYN)	Emotional Exhaustion (Emo_Exha)	Professional Efficacy (Prof_Eff)
Cynicism (CYN)		B = 0.533 *p* ˂ 0.001 ES = 0.088	β = −0.119 *p* = 0.046 ES = 0.07
Emotional Exhaustion (Emo_Exha)	β = 0.116 *p* = 0.05 ES = 0.070		β = −0.224 *p* = 0.011 ES = 0.096
Professional Efficacy (Prof_Eff)	β = −0.519 *p* ˂ 0.001 ES = 0.089	β = −0.277 *p* ˂ 0.001 ES = 0.067	
Normal Weight (BMI)	β = −0.147 *p* = 0.019 ES = 0.07	β = −0.355 *p* ˂ 0.001 ES = 0.093	β = 0.283 *p* = 0.002 ES = 0.095

**Table 8:** Latent variable validation—Overweight model.

Coefficient	Cynicism (CYN)	Emotional Exhaustion (Emo_Exha)	Professional Efficacy (Prof_Eff)	Overweight (BMI)
R-Squared	0.298	0.026	0.111	0.014
Adj. R-squared	0.294	0.020	0.106	0.008
Composite Reliability	0.923	0.938	0.908	1
Cronbach’s Alpha	0.888	0.918	0.877	1
Average Variance Extracted	0.750	0.753	0.623	1
Full Collinearity VIFs	1.449	1.421	1.042	1.018
Q-Squared	0.302	0.028	0.109	0.016

**Table 9:** Model fit indices—Overweight model.

Model Fit Index	Value	Decision Criteria
Average Path Coefficient (APC)	0.29	*p* < 0.001
Average R-Squared (ARS)	0.112	*p* = 0.034
Average Adjusted R-Squared (AARS)	0.107	*p* = 0.039
Average Block VIF (AVIF)	1.214	Acceptable ≤ 5, ideal ≤ 3.3
Average Full collinearity VIF (AFVIF)	1.232	Acceptable ≤ 5, ideal ≤ 3.3
Tenenhaus GoF (GoF)	0.296	Small ≥ 0.1, medium ≥ 0.25, large ≥ 0.36

**Table 10:** Effects size—Overweight model.

TO	FROM			
	Cynicism (CYN)	Emotional Exhaustion (Emo_Exha)	Professional Efficacy (Prof_Eff)	Overweight (BMI)
Cynicism		0.298		
Emotional Exhaustion (Emo_Exha)			0.026	
Professional Efficacy (Prof_Eff)	0.111			
Overweight (BMI)	0.014			

**Table 11:** Sum of indirect effects—Overweight model.

TO	FROM		
	Cynicism (CYN)	Emotional Exhaustion (Emo_Exha)	Professional Efficacy (Prof_Eff)
Cynicism (CYN)		β = −0.088 *p* = 0.05 ES = 0.017	
Emotional Exhaustion (Emo_Exha)	β = 0.054 *p* = 0.158 ES = 0.029		
Professional Efficacy (Prof_Eff)	β = −0.182 *p* < 0.001 ES = 0.023	β = 0.029 *p* = 0.253 ES = 0.029	
Overweight (BMI)	β = −0.065 *p* = 0.114 ES = 0.07	β = 0.010 *p* = 0.406 ES = 0.000	

**Table 12:** Total effects—Overweight model.

TO	FROM		
	Cynicism (CYN)	Emotional Exhaustion (Emo_Exha)	Professional Efficacy (Prof_Eff)
Cynicism (CYN)		β = 0.546 *p* ˂ 0.001 ES = 0.298	β = −0.088 *p* = 0.050 ES = 0.017
Emotional Exhaustion (Emo_Exha)	β = 0.054 *p* = 0.158 ES = 0.029		β = −0.162 *p* = 0.015 ES = 0.026
Professional Efficacy (Prof_Eff)	β = −0.333 *p* ˂ 0.001 ES = 0.111	β = −0.182 *p* ˂ 0.001 ES = 0.023	
Overweight (BMI)	β = −0.119 *p* = 0.057 ES = 0.014	β = −0.065 *p* = 0.114 ES = 0.007	β = 0.010 *p* = 0.406 ES = 0.000

**Table 13:** Latent variable validation—Obesity model.

Indices	Cynicism (CYN)	Emotional Exhaustion (Emo_Exha)	Professional Efficacy (Prof_Eff)	Obesity (BMI)
R-Squared	0.371	0.129	0.132	0.043
Adj. R-Squared (ARS)	0.364	0.120	0.123	0.022
Composite Reliability	0.875	0.939	0.913	1.000
Cronbach’s Alpha	0.807	0.918	0.885	1.000
Average Variance Extracted	0.641	0.754	0.636	1.000
Full Collinearity VIFs	1.649	1.603	1.139	1.021
Q-Squared (Q²)	0.372	0.133	0.127	0.049

**Table 14:** Model fit indices—Obesity model.

Indices	Value	Decision Criteria
Average Path Coefficient (APC)	0.326	*p* < 0.001
Average R-Squared (ARS)	0.169	*p* = 0.034
Average Adjusted R-Squared (AARS)	0.157	*p* = 0.039
Average Block VIF (AVIF)	1.002	Acceptable ≤ 5, ideal ≤ 3.3
Average Full Collinearity VIF (AFVIF)	1.353	Acceptable ≤ 5, ideal ≤ 3.3
Tenenhaus GoF (GoF)	0.358	Small ≥ 0.1, medium ≥ 0.25, large ≥ 0.36

**Table 15:** Effects size—Obesity model.

TO	FROM			
	Cynicism (CYN)	Emotional Exhaustion (Emo_Exha)	Professional Efficacy (Prof_Eff)	Obesity (BMI)
Cynicism		0.371		
Emotional Exhaustion			0.129	
Professional Efficacy	0.132			
Obesity		0.022	0.021	

**Table 16:** Sum of indirect effects—Obesity model.

TO	FROM		
	Cynicism (CYN)	Emotional Exhaustion (Emo_Exha)	Professional Efficacy (Prof_Eff)
Cynicism (CYN)			β = 0.219 *p* < 0.001 ES = 0.070
Emotional Exhaustion (Emo_Exha)	β = 0.131 *p* = 0.032 ES = 0.070		
Professional Efficacy (Prof_Eff)		β = −0.222 *p* ˂ −0.001 ES = 0.068	
Obesity (BMI)	β = −0.073 *p* = 0.151 ES = 0.071	β = −0.033 *p* = 0.289 ES = 0.058	β = 0.054 *p* = 0.223 ES = 0.008

**Table 17:** Total effects—Obesity model.

TO	FROM		
	Cynicism (CYN)	Emotional Exhaustion (Emo_Exha)	Professional Efficacy (Prof_Eff)
Cynicism		β = 0.609 *p* < 0.001 ES = 0.086	β = −0.219 *p* < 0.001 ES = 0.068
Emotional Exhaustion	β = 0.131 *p* = 0.032 ES = 0.070		β = −0.359 *p* < 0.001 ES = 0.092
Professional Efficacy	β = −0.364 *p* < 0.001 ES = 0.092	β = −0.222 *p* < 0.001 ES = 0.068	
Obesity	β = −0.073 *p* = 0.151 ES = 0.071	β = −0.184 *p* = 0.030 ES = 0.097	β = 0.202 *p* = 0.020 ES = 0.097
